# Unusual Suspects: The People Inside and Outside of School Who Matter in Whole School, Whole Community, Whole Child Efforts

**DOI:** 10.1111/josh.12966

**Published:** 2020-11-12

**Authors:** Karen Pittman, Deborah A. Moroney, Merita Irby, Jill Young

**Affiliations:** ^1^ Forum for Youth Investment, 7064 Eastern Avenue, NW Washington DC 20012; ^2^ American Institutes for Research, 10 S Riverside Plaza, Ste 600 Chicago IL 60606

**Keywords:** learning and development, whole child, WSCC model

## Abstract

**BACKGROUND:**

Individuals learn and develop across their lifespan in response to the people with whom they interact in settings, communities, cultures, and experiences. Collaborative research and policy efforts underscore the critical and reparative role of identity‐safe, culturally driven, and relationship‐rich environments—in classrooms, schools, and communities—for the healthy social, emotional, cognitive, and physical development of all learners. These themes align closely with the Whole School, Whole Community, Whole Child (WSCC) model.

**METHODS:**

Collaborative research and policy efforts, combined with the WSCC model make a strong case to elevate the unusual suspects, the settings and people outside of instructional classrooms who are key actors in creating the conditions and relationships that promote student health and well‐being.

**RESULTS:**

We describe promising cases of efforts aimed at bolstering adult capacity to foster student health and well‐being. We also discuss how these types of systemic efforts can move beyond system and program level actors to acknowledge the power of individual‐level implementation.

**CONCLUSIONS:**

It is time to recognize the systemic efforts, programmatic opportunities, and individual roles adults have in implementing an ideal WSCC model that is aligned with the collaborative research and policy efforts.

Learning and development are integrated, ongoing processes.^1^, ^2^ Individuals learn and develop across their lifespan in response to the people with whom they interact in settings, communities, cultures, and experiences.^1^, ^2^ Early childhood and adolescence are times for heightened learning and development.^3^, ^4^ Schools can play a central coordinating role in learning and development that reaches well beyond their academic charge by promoting the long‐term development and success of all children and youth.^5^

The Whole School, Whole Community, Whole Child (WSCC) model speaks directly to the need to coordinate supports for growth and learning. The WSCC model emphasizes coordinated policies and practices to optimize social and emotional climate, physical environment, and family and community involvement.^5^ The WSCC model aligns well with the findings of several collaborative research and policy efforts that have summarized the convergent science base on learning, development, and thriving. These collaborative research and policy efforts describe the tenets of a student‐centered approach (whole child), include as a primary focus the implications of this approach for the K‐12 system (whole school) and, to varying degrees, explore the importance of this approach for the settings and systems where children and youth spend their time outside of school (whole community).

The collaborative research and policy efforts support what developmentalists have long argued—a young person's learning and development is shaped in positive and negative ways by both the simple and the sustained interactions they have with the adults in their life.^1^ This includes adults they engage with on a regular basis, such as teachers, parents, and caregivers, youth development leaders, coaches, teaching artists, and safety officers. This also includes adults they may interact with in more targeted ways—the unusual suspects or unsung heroes—such as librarians, counselors, social workers, healthcare professionals, case managers, court advocates, and museum docents.

The press to strengthen the knowledge and capacity of adults to create relationship‐rich environments that support learning and development is already happening within the education system and within the field of youth development. The WSCC model closely aligns with this idea, specifically with the addition of model components, such as social and emotional climate, physical environment, and community involvement, and the emphasis on coordinated policy, process, and practice.^5^ Combined, the findings on the importance of relationships and context and the WSCC model can be used to make a strong case to elevate the unusual suspects, or the settings and people outside of instructional classrooms who are key actors in creating the conditions and relationships that promote student health and well‐being.

In this paper, we explore the WSCC model in conjunction with recent collaborative research and policy efforts driving systemic efforts to build equitable and promotive conditions for learning and development that support student health and well‐being. We also discuss how systemic efforts can move beyond system and program level actors to acknowledge the power of individual‐level implementation.

## Literature Review

Collaborative research and policy efforts include the Science of Learning and Development (SoLD) Alliance's core papers and initial findings report;^1^, ^2^ the National Academies of Science, Engineering, and Mathematics (NASEM) report *Promise of Adolescence: Realizing Opportunity for All Youth*; the National Commission of Social, Emotional, and Academic Development (SEAD Commission) report *From a Nation At Risk to a Nation At Hope*;^3^ and the Alliance for Excellent Education's (All4Ed) report *Science of Adolescent Learning*.^4^ We highlight these collaborative research and policy efforts because they integrate the emerging cross‐discipline findings from neuroscience, psychology, sociology, and more, and contribute to a powerful body of work informing learning and development.^1^, ^2^ The convergence of collaborative research and policy efforts on learning, development, and thriving creates an opportunity to forge a common understanding of the developmental practices that must be used consistently in the places, systems, and settings where children and youth spend their time. Like the WSCC model, these efforts highlight the pivotal role all adults play in all settings in supporting young people's well‐being and ability to thrive. In the following paragraphs, we describe some of the collaborative research and policy efforts and connect them to the WSCC model.

The SoLD Alliance's syntheses of science across multiple disciplines emphasize several findings:^1^, ^2^

- The malleability of the brain, especially in adolescence;
- The critical and reparative role of identity‐safe, culturally driven, and relationship‐rich environments for the healthy development of all learners;
- The individuality of learning paths and unevenness of skill development; and
- The connection between cognitive, social and emotional development and the ability to make meaning of experiences and perform complex actions.

The SoLD Alliance's framing is particularly relevant for school leaders. It highlights the importance of understanding the cumulative and immediate effect each experience can have on the next and underscores the value of optimizing every environment. These findings align well with the WSCC model's focus on settings and relationships beyond the academic classroom. These include specific settings and adult relationships associated with nutrition, physical education and activity, counseling and social services, health services as well as the overall physical environment and social and emotional climate.^6^ The SoLD Alliance findings suggest the need to elevate the importance of youths' relationships and experiences with adults associated with these specific non‐academic services and with adults assigned to non‐academic settings, such as cafeterias, playgrounds, extracurricular clubs, and teams.

The SEAD Commission 2018 report, *From a Nation At Risk to a Nation At Hope*, offers 6 recommendations that align closely with the WSCC model. The first recommendation—“Set a clear vision that broadens the definition of student success to prioritize the whole child”^6^—clearly mirrors the goals of the WSCC model (students who are healthy, safe, engaged, supported, and challenged). The fifth recommendation hones in on the adults, partnerships, and experiences in spaces outside of the classroom and school: “Align resources and leverage partners in the community to address the whole child.”^6^ The remaining recommendations from the SEAD Commission focus on the need to build adult capacity and create supportive, challenging learning environments. Indeed, the idea of school, community, and program partnerships is not a new one, nor is supporting adult capacity. But we now have an increasingly convergent science base and a “hopeful” policy charge to assert these settings matter in creating the conditions for learning and development. The adults in all of the spaces, in school and out, are not only critical, but are non‐negotiable actors in ensuring all young people have the opportunity to thrive.

Also, as part of the SEAD Commission, the Youth Development Work Group elevated the roles that the range of adults play no matter what the setting. This includes community organizations; the leaders and staff in these organizations often have relationships with young people families, and other community partners that facilitate trust building and engagement.^7^ For example, the report called for a “shared understanding of where and when learning happens that maximizes the use of all the places where young people spend their time, from schools to community organizations and from the home to the workplace—all of which have the potential to support social, emotional, cognitive, and academic learning.”^7^ The WSCC model also highlights the need for greater alignment, integration, and coordination between schools, families, health services, and community members and organizations to improve each student's cognitive, physical, social, and emotional development.^5^

The All4Ed 2018 report *Science of Adolescent Learning* highlights findings about how adolescents learn and develop and share recommendations for how educators, policymakers, and advocates can apply the findings to policy and practice.^4^ For example, the report recommends, “District and school leaders should design organizational structures, including academic support systems, school improvement efforts, structures that foster positive relationships, and wraparound services that respond to the learning and developmental needs of adolescent learners, supporting their academic, social, emotional, physical, and health needs.”^4^ This recommendation aligns with the WSCC model, which is “directed at the whole school, with the school in turn drawing its resources and influences from the whole community and serving to address the needs of the whole child.”^4^

NASEM's 2019 report *Promise of Adolescence: Realizing Opportunity for All Youth* underscores the importance of pressing school leaders to revisit policy and practice decisions based on widely held assumptions about adolescents and adolescent development.^3^ It goes beyond K‐12 education to call for this information to be used to create a cross‐system view of adolescent development, which brain science demonstrates extends into the mid‐twenties.^3^ Also grounded in the science of adolescence, the report explores implications for not only schools, but also for other youth‐serving systems, including health, child welfare, and justice. The report acknowledges the extent to which adults and young people traverse those system boundaries and recognizes the need for cross‐system collaboration and alignment. It does not, however, offer specific recommendations. The WSCC model takes this step. It explicitly encourages coordination of policies, processes, and practices across the academic and non‐academic administrative units within school systems.

## The WSCC Model in Action

Each of the 4 consensus reports had a different charge. Combined, they create a set of arguments for expanding efforts to create coordinated and integrated approaches for learning and healthy development, in academic and non‐academic settings, in schools and communities for children and adolescents.

Now, we provide 3 promising cases of practice efforts that illustrate the WSCC model in action. We highlight 3 organizations that work directly with school systems and school personnel to bring the convergent science findings into practice: Playworks, Partnership for Children & Youth, and City Year.

## Playworks

Playworks helps students stay active while building social and emotional skills through play.^8^ The organization supports schools and districts to improve play and recess by supporting on‐site staffing and providing consultative support, professional development, and free resources. The organization also supports youth programs and other organizations to improve playtime. Playworks take a holistic approach to ensure that recess reinforces school culture, focusing on safety, engagement, and empowerment. Schools that partner with Playworks have a recess team composed of various school stuff such as administrators, teachers, and paraprofessionals, and a recess coach who facilitates recess at the school. Schools also have a junior coach, often fourth or fifth grade students, who receive team‐building training. Finally, there are Playworks site coordinators, who partner with 4 schools and spend a week per month with each school to lead, support, and empower the school recess team to create a safe and inclusive recess for all students. An impact study identified several positive findings for schools with Playworks programs compared to schools without: (1) students are more physically active at Playworks schools; (2) schools have less bulling; (3) teachers gain valuable time transitioning from recess to the classroom; (4) students experience increased safety; (5) and students are more attentive in class.^9^

Playworks wanted to learn how recess team member attitudes, skills, behaviors, and character traits relate to the quality of Playworks implementation. Findings from their evaluation indicated that Playworks is most successful when the adults who facilitate recess value play and support Playworks.^10^ The evaluation results also highlighted the critical role of school administrators in facilitating quality recess—recess teams without an engaged school administrator perceived less schoolwide support for Playworks and reported more implementation challenges.^10^ Finally, the evaluation results indicated that teacher buy‐in is key to successful Playworks implementation, but teachers are not often included on recess teams and may not know as much about Playworks programs.^10^

Playworks is an example of physical education and physical activity aligned with the WSCC model. The WSCC model's physical education and physical activity component advocates for schools to create an environment that offers several opportunities to be physical active throughout the school day.^11^ Physical education programs provide opportunities to develop “skills, knowledge, and behaviors for healthy active living, physical fitness, sportsmanship, self‐efficacy, and emotional intelligence.”^11^ The WSCC model calls for strong coordination across 5 components: physical education, physical activity during school, physical activity before and after school, staff involvement, and family and community engagement.^11^ The results of the Playworks evaluation underscore the WSCC's guidance on strong coordination for physical education and activity. Playworks found that, unsurprisingly, both principals and teachers are partners in successful implementation and support for play during non‐instructional time. The study findings highlight the key role of all adults in the school building play in physical education and physical activity, not just those directly responsible for recess.

## Partnership for Children and Youth

Partnership for Children and Youth launched an initiative called Expanded Learning 360/365 in 2015 in response to what the organization recognized as an overlooked opportunity to align capacity‐building efforts to strengthen social and emotional learning (SEL) practices of adults who work with youth in school, afterschool, and summer learning settings. Partnership for Children and Youth is an advocacy and capacity‐building organization championing high‐quality learning opportunities for underserved youth in California, with an emphasis on afterschool, summer learning, and community schools.^12^ Partnership for Children and Youth work with people, organizations, and systems to leverage their existing resources and work together to educate and support children in under‐resourced communities. The organization does this by training program providers, facilitating relationships between school districts and community‐based organizations, and advocating for public policies.

Both school districts and expanded learning programs demonstrated a growing interest in SEL, but they were not working together to achieve common goals. Partnership for Children and Youth created professional learning communities to address cross‐sector (in school and out‐of‐school) alignment on SEL and school climate. The initiative brought together leaders from school districts and expanded learning organizations to focus on planning, aligning, and implementing SEL across the school day, afterschool, and summer.

An evaluation of the initiative identified 3 strengths. First, the initiative provided an inclusive and structured environment that fostered trust, respect, and collective responsibility among members.^13^ Second, the initiative provided dedicated time for the group to authentically align SEL strategies between the school day and extended learning organizations.^13^ Finally, the initiative created space for implementation strategies, such as group and individual reflection, action planning, and continuous improvement.^13^

Partnership for Children and Youth's Expanded Learning 360/365 initiative represents an example of the social and emotional school climate component of the WSCC model. Social and emotional climate focuses on “the psychosocial aspects of students' educational experience that influence their social and emotional development.”^11^ The social and emotional climate of a school can impact a broad array of outcomes: student engagement in school activities; relationships with other students, staff, family, and community; and academic performance.^11^ A positive social and emotional school climate promotes “health, growth, and development by providing a safe and supportive learning environment.”^11^ Partnership for Children and Youth recognized an opportunity for greater alignment, integration, and collaboration between school districts and expanded learning organizations to improve SEL. This initiative allowed them to align common goals across 2 sectors to focus more holistically on young people's social and emotional development. The initiative provides an example of a way to bolster school day supports for SEL through collaborative partnerships with expanded learning organizations.

## City Year

City Year is a national program that provides support to high schools, zooming in on the need to use the *Science of Adolescent Learning*.^14^ City Year developed a Whole School Whole Child approach to support student and school success. City Year's model, like the WSCC model, recognizes that students' holistic needs should be valued and addressed by adults throughout the school community. The organization aims to provide a holistic approach to support individual student, classroom, and whole school needs. City Year does this by providing additional support and capacity to create positive schoolwide learning environments. The organization collaborates with district leaders to identify schools that would most benefit from AmeriCorps members, and then places members in these schools to implement their Whole School Whole Child model. AmeriCorps members are fully integrated into schools as full‐time staff members. They serve all students in a school and provide additional support to students at risk of dropping out of school.

City Year's Whole School Whole Child model focuses on academic, social, and emotional services aimed at helping students develop the skills they need to navigate and complete high school. The organization has developed and widely implemented 2 tiers of Whole School Whole Child services: universal and targeted. Universal services are those provided to all students in a school. Services include afterschool programs and schoolwide events that recognize students' positive behavior and success. Services also include in‐classroom support for English language arts and math teachers. Targeted services are provided to students who are at increased risk of not graduating. The at‐risk determination is based on early warning indicators such as attendance, behavior, and performance in English language arts or math. Services in this tier include tutoring, social and emotional development and behavior support, and attendance coaching.

City Year has studied the impact of its Whole School Whole Child approach. One study found that schools that partnered with City Year were more than 2 times more likely to improve proficiency rates in English Language Arts and up to 3 times more likely to improve proficiency rates in math than schools that did not partner with City Year.^15^ In addition, schools that partnered with City Year gained the equivalent of approximately 1 month of additional math and English Language Arts learning compared with non‐City Year schools.^15^ City Year currently has an evaluation underway to examine the impact and implementation of their Whole School Whole Child model for middle schools. The organization recognizes the middle school as a pivotal time for students to form identities and learn the academic, social, and emotional skills to succeed in life. The results of the evaluation will build evidence on what works for middle school students in their pathway to high school graduation. The results of the evaluation will also contribute to the evidence base on the impact of whole child approaches.

Each of these organizations, Playworks, Partnership for Children and Youth, and City Year, has taken on efforts that, intentionally or not, embody the WSCC model or components of the model. One common thread across these examples is the importance of strong coordination of the adults who work with young people. For example, the Playworks found their model for supporting physical education and physical activity to facilitate skill development worked best when multiple members of the school building were involved. Partnership for Children and Youth dedicated resources and opportunities to connect schools and expanded learning organizations so they could coordinate and align their SEL goals. City Year developed universal and targeted services based on their own Whole School Whole Child model, which closely aligns with the WSCC model, to better support school personnel and students. The examples provided demonstrate that the WSCC model and other whole child approaches can be more than an aspirational goal by showing us what the model and its components look like in the real world.

## Unsung Champions, Unsung Roles, and Unsung Settings

The collaborative research and policy efforts and the practice efforts along with the WSCC model underscore the essential role of all adults in all settings—when, where and with whom learning happens. In this section, we recognize and describe efforts from adults who are unsung champions in unsung settings.

Collaborative research and policy efforts are shifting the definition of optimized learning from a focus on content transmission to a focus on learner transformation—on supports for the development of portable skillsets and mindsets that give young people a sense of competence, agency, and identity.^6^, ^16^ If every experience and interaction that a young person has matters, the adults involved in shaping those experiences matter, regardless of the setting—classroom, playground, gym, library, cafeteria, bus, health room, or afterschool or summer program.

In 2014, non‐teaching staff comprised half of the public school workforce.^17^ The number of non‐teaching staff in schools has grown by 130% over the past several decades.^17^ This increase reflects both the increased responsibilities schools have taken on to mainstream students with disabilities and provide extra academic supports to students in need and also increased responsibilities for transportation, meals, health and basic services and before and after school programs.^13^ Schools have also used partnerships to increase the numbers of adults involved in supporting their students before, during, and after school.^7^, ^18^

The potential to provide more and better‐integrated support for student learning and healthy development takes more than increased numbers. In our work, we have observed that efforts to ensure that all adults connected to school have a sense of shared accountability for the social, emotional, cognitive, and physical well‐being of students in all the learning settings where they spend time can be a multiplier.

For example, a 2019 study of school nutrition programs delivering breakfast in the classroom highlighted an unanticipated yet common theme of conversations with parents and staff: a more nurturing start to the school day was a clear value‐add with social and emotional benefits in addition to the anticipated physical benefits.^19^ In another example, a 2012 study of a school‐based mentoring program found that mentoring relationship quality, not just the access to a mentor, was significantly associated with positive changes in youths' relationships with parents and teachers. Higher quality relationships with parents and teachers, in turn, were significantly associated with better youth outcomes, including self‐esteem, academic attitudes, prosocial behaviors, and misconduct.^20^

## IMPLICATIONS FOR SCHOOL HEALTH

These and other studies demonstrate that a range of caring and connected adults who cross boundaries between school and community are key to powerful, front‐line connections. There are unsung heroes in every school. These professionals serve as unrecognized ambassadors with the potential to carry great power in a WSCC model if they are recognized and supported in their boundary crossing role(s). In the end, each adult in a young person's space carries great power and a responsibility to provide identity‐safe, developmentally rich experiences and relationships that ensure all young people can thrive. The adults who work with children and youth across systems and settings would be doubly empowered if they have:

- A deeper understanding of the diverse assets and starting points of young people, the richness of their communities and cultures, the barriers and challenges they face, and the totality of places where they spend their time.
- A common understanding of what “good” looks like when it comes to creating the conditions for successful learning and development. This is true not only for adults working in different settings, but also for adults working within the same system that come from different disciplines and professions, such as teachers, counselors, safety officers, cafeteria workers, and health aides.
- A greater appreciation of the variation in the roles they play and the settings they work in and the differences in the opportunities for interaction and instruction they can create because of these differences.
- Regular opportunities to connect across disciplines, systems and settings—sharing not only knowledge of what works but, especially at the community level, building connections and sharing responsibility for the success of each individual young person.

The adults working directly with young people are the alchemists who have the power and ability to create the environment in which young people learn and thrive. Adults are the climate‐setters. In each setting and situation, the adults in the room, on the playground, or in the park—the educators and practitioners on the front‐line—have an opportunity to create the climate to support each young person's health and well‐being.

In this paper, we described the findings from collaborative research and policy efforts and how they connect to the WSCC model. We also highlighted organizations who put the WSCC model components into practice. Finally, we described the essential role all adults play healthy learning and development. It is time to recognize the systemic efforts, programmatic opportunities, and individual roles adults have in implementing an ideal WSCC model that is aligned with the collaborative research and policy efforts.
